# Machine Learning: A New Prospect in Multi-Omics Data Analysis of Cancer

**DOI:** 10.3389/fgene.2022.824451

**Published:** 2022-01-27

**Authors:** Babak Arjmand, Shayesteh Kokabi Hamidpour, Akram Tayanloo-Beik, Parisa Goodarzi, Hamid Reza Aghayan, Hossein Adibi, Bagher Larijani

**Affiliations:** ^1^ Cell Therapy and Regenerative Medicine Research Center, Endocrinology and Metabolism Molecular-Cellular Sciences Institute, Tehran University of Medical Sciences, Tehran, Iran; ^2^ Diabetes Research Center, Endocrinology and Metabolism Clinical Sciences Institute, Tehran University of Medical Sciences, Tehran, Iran; ^3^ Endocrinology and Metabolism Research Center, Endocrinology and Metabolism Clinical Sciences Institute, Tehran University of Medical Sciences, Tehran, Iran

**Keywords:** artificial intelligence, cancer, data analysis, machine learning, multi-omics

## Abstract

Cancer is defined as a large group of diseases that is associated with abnormal cell growth, uncontrollable cell division, and may tend to impinge on other tissues of the body by different mechanisms through metastasis. What makes cancer so important is that the cancer incidence rate is growing worldwide which can have major health, economic, and even social impacts on both patients and the governments. Thereby, the early cancer prognosis, diagnosis, and treatment can play a crucial role at the front line of combating cancer. The onset and progression of cancer can occur under the influence of complicated mechanisms and some alterations in the level of genome, proteome, transcriptome, metabolome etc. Consequently, the advent of omics science and its broad research branches (such as genomics, proteomics, transcriptomics, metabolomics, and so forth) as revolutionary biological approaches have opened new doors to the comprehensive perception of the cancer landscape. Due to the complexities of the formation and development of cancer, the study of mechanisms underlying cancer has gone beyond just one field of the omics arena. Therefore, making a connection between the resultant data from different branches of omics science and examining them in a multi-omics field can pave the way for facilitating the discovery of novel prognostic, diagnostic, and therapeutic approaches. As the volume and complexity of data from the omics studies in cancer are increasing dramatically, the use of leading-edge technologies such as machine learning can have a promising role in the assessments of cancer research resultant data. Machine learning is categorized as a subset of artificial intelligence which aims to data parsing, classification, and data pattern identification by applying statistical methods and algorithms. This acquired knowledge subsequently allows computers to learn and improve accurate predictions through experiences from data processing. In this context, the application of machine learning, as a novel computational technology offers new opportunities for achieving in-depth knowledge of cancer by analysis of resultant data from multi-omics studies. Therefore, it can be concluded that the use of artificial intelligence technologies such as machine learning can have revolutionary roles in the fight against cancer.

## Introduction

Cancer is categorized as one of the pre-eminent causes of human fatality throughout the world (Tayanloo-Beik et al., 2020). According to the latest data released by International Agency for Research on Cancer (IARC), the incidence and mortality of cancer are estimated at 19.3 million and 10.0 million people, respectively by 2020. But what adds to the importance of this issue is that if preventive measures are not implemented, the incidence of cancer will reach 28.4 million people by the next 20 years (Sung et al., 2021). Hence, it can be understood that cancer is a serious problem that human beings struggle with and can have adverse effects at the level of individuals or communities. In addition, the cancer issue can have significant impacts on government entities such as the healthcare and economic systems (Wild, 2019). Accordingly, achieving the deep knowledge of mechanisms underlying cancer on a small cell scale can be a big step towards early prediction, detection, and treatment of cancer and eventually would have astonishing effects at the global level (Golemis et al., 2018). In cellular and molecular studies, cancer is defined as a broad range of diseases in which the cells are capable to grow and divide in an uninhibited manner and some cases tend to impinge on other tissues and organs through metastasis that can affect almost any part of the body (Tayanloo-Beik et al., 2020). Evidence has documented that the onset and progression of cancer are not at once. Instead, the characteristics of the cancerous cells manifest in a step-by-step process (Frank, 2018). These steps represent the accumulation of molecular changes over a long period of time in DNA, RNA, proteins, metabolites, and so on (Chakraborty et al., 2018). Since the molecular alterations can affect many layers of cell biology, in recent years the field of omics science and related technologies have made a great contribution to learn the rope of mechanisms underlying the onset and progression of cancer. While the single-layer-omics study have been able to provide researchers with a deeper insight of view on the identity of cancer, the complexities of genotype-phenotype-environmental interactions behind cancer can be achieved through the integration of different branches of omics as a multi-omics field in cancer biology. Additionally, multi-omics-based studies can pave the way for examining various aspects of cancer such as investigation of cellular responses to treatments and exploration of novel prognostic, diagnostic, and therapeutic approaches in cancer management (Menyhárt and Győrffy, 2021). Accordingly, the study of cancer on such a large scale is associated with a data explosion (Chakraborty et al., 2018). To access the related data, a variety of online resources in the field of cancer research in combination with the multi-omics arena are available to provide a comprehensive understanding of molecular patterns involved in cancer at the level of genome, transcriptome, proteome, metabolome, and so on (Das et al., 2020). The existence of big data in the field of multi-omics studies requires elaborate computational analysis. Therefore, in light of rapid advancement in the development of technology, the field of artificial intelligence (AI) and the related subsets particularly machine learning (ML) have been able to leap forward into assisting the analysis of the big data resultant multi-omics studies (Moezzi et al., 2021) in tackling different diseases such as cancer (Nicora et al., 2020). ML, one of the branches of AI, can be used as a computational tool which attempts to parse input data, recognize patterns, and ultimately provide some knowledge in the output (Nagy et al., 2020). In recent years, some advances are being made through the collaborations between ML and multi-omics data analysis of cancer which primary intent is to provide a broad view of the complexities of the patterns involved in the cancer process (de Anda-Jáuregui and Hernández-Lemus, 2020). To promote research in the field of ML application in multi-omics data analysis of cancer, this review initially provides an overview of understanding the different aspects of cancer biology while pointing out current methods applied in cancer management. Then, the role of the multi-omics arena in combination with oncology studies are highlighted. Subsequently, recent advances in the application of ML methods in combination with cancer omics data assessments are particularly described. Finally, several challenges of applying ML in omics data analysis and some effective solutions to address these challenges are described.

## An Overview of Cancer

The biology of cancer is composed of highly sophisticated intracellular, intercellular, intertissular, and intersystem interplays in a step-by-step process at a level of an organism (Paul, 2020). In order to promote the perception of cancer process, this is urgent to zoom in and examine each step of this process separately. Although, various categorize have been considered for the steps of cancer development, in this review, carcinogenesis is scrutinized in three general and particular stages as detailed below (Weston and Harris, 2003):

1) Initiation: According to the last researches, cancer risk factors can be generally divided into two groups: Intrinsic and non-intrinsic risk factors. The intrinsic factors have been attributed to unpredictable random spontaneous mutations which can take place during the DNA replication process. In contrast, the non-intrinsic risk factors can be categorized as two endogenous and exogenous factors that contributed to carcinogenesis. In a more detailed study of the mentioned two subgroups, the endogenous risk factors such as the function of hormones, immune and metabolic systems, or the genetic susceptibility particularly depend on every individual’s biological characteristics. Conversely, different factors related to an individual’s lifestyle such as diet, obesity, and some viral infections or environmental agents like exposure to chemicals and hazardous radiations can be classified as exogenous risk factors (Wu et al., 2018). All of these categories and more on can play a key role in initiation of carcinogenesis multistep process by occurrence of genetic mutations and epigenetic alterations (Belizário, 2018; Sheikh-Hosseini et al., 2021). In other words, the epigenome and the genome can have mutual interaction in the onset of cancer. Indeed, genetic mutations in epigenome-related sequences can lead to epigenetic changes, and contrariwise, epigenomic alterations can lead to mutations in DNA which both have great contribution in cancer initiation (You and Jones, 2012). More detailed evaluation findings imply that two groups of genes, proto-oncogenes and tumor suppressor genes, are affected by genetic and epigenetic alterations. Thus, risk factors can have a major contribution in genomic or epigenomic alterations, which in turn adjust the expression and the function of proto-oncogenes and tumor suppressor genes. Accordingly, any dysfunction of tumor suppressor genes or overexpression of proto-oncogenes can trigger the cancer promotion step (Wang et al., 2018).
2) Promotion: Once the cells are affected by the initiators factors and the accumulation of alterations is enough to onset the cancer, the initiated cells transit to “promotion” step. At this step, the most important phenomenon is that initiated cells are susceptible to the effects of promoter’s agents. One of the functional characteristics of promoter agents is that they indirectly affect genes. Indeed, unlike initiator factors, they are non-mutagenic (Weston and Harris, 2003). Functionally, they can cause alterations in different intracellular processes such as cell signaling, gene expression, apoptosis, etc. by interaction with cellular receptors. Hence, the cells would be triggered to grow and proliferate in an uncontrolled manner. Consequently, the cells expanded into a colony and the population of cancer cells increased (Mendelsohn et al., 2014).
3) Progression and metastasis: The final step of cancer refers to the irreversible transformation of a benign tumor or pre-neoplasm into a malignant tumor or neoplasm. At this step the genome is highly unstable. Consequently, the cells are susceptible to be affected by more genetic and epigenetic alterations. These changes can be accompanied by hyperactivation of proto-oncogenes and hypoactivation or loos of function of tumor suppressor genes (Weston and Harris, 2003; Mendelsohn et al., 2014). Additionally, karyotypic variations, aneuploidy (Sansregret and Swanton, 2017), and polyploidy are observed at this step (Baudoin and Bloomfield, 2021). As the disease progresses, the cells would have a strong tendency to separate from the colony and impinge to other tissues in the body through different mechanisms underlying the metastasis process. Hence to disperse the cells from the primary tumor, various interactions between cancer cells and their microenvironments such as alterations in the function of cell junctions and cell adhesions molecules or the initiation of epithelial-mesenchymal transition, angiogenesis, and lymphangiogenesis can be the subsequent phenomenons in the progression of cancer. As a result, the cells can easily detach from the cancerous cells population and migrate to other parts of the body through bloodstream (Martin et al., 2013).

Therefore, according to the alterations of mechanisms underlying cells during the onset to development of cancer, as mentioned above, cancer cells acquire several biological features, including 1) Genomic instability and chromosomal abnormalities, 2) Indefinite growth and uncontrolled proliferation potential, 3) Replicative immortality, 4) Evading growth suppressors and cell death signaling, 5) Remodeling the extracellular matrix and forming rich and dynamic tumor microenvironment, 6) Evading immune surveillance and destruction, 7) Reprograming cellular energetics, 8) Inducing tumor-promoting inflammation, 9) Activating the angiogenic switch, 10) Enabling invasion and metastasis, 11) Alteration in microbiome, which are all known as the hallmarks of cancer (Fouad and Aanei, 2017; MacCarthy-Morrogh and Martin, 2020).

## Current Methods and Techniques in Cancer Management

Due to the increasing global cancer incidence rate, any urgent and prompt prevention and management actions can be an obstacle to the growing trend of cancer. Consequently, in addition to significantly increasing the survival rate, the quality of life of the patients can be improved (Tobore, 2019). Hence, for a more detailed study of current methods and techniques in cancer scrutiny, in this review, the cancer management strategies have been categorized into four levels of prevention, early detection and diagnosis, treatment, and palliative care according to the World Health Organization (WHO) guide for effective cancer control programs that are detailed below (Organization, 2007).

### Cancer Prevention

In a general category, cancer prevention can be examined in three ways, including primary, secondary, and tertiary (Wray et al., 2018). Primary prevention of cancer is a stage in which the occurrence of the disease can be obstructed as much as possible due to applying interventions and efforts at two individual and community levels. As mentioned earlier, genetic, environmental factors or the integration of both can be the origin of the different types of cancers. Therefore, taking any measures to reduce or eliminate cancer risk factors [e.g., tobacco, alcohol consumption, dietary habits especially inadequate consumption of fruits and vegetables, high body mass index (BMI), inadequate physical activity, reproductive factors, and types of carcinogens] can be an effective step in preventing cancer (Organization, 2008).

In addition, preventive interventions at the community level are mainly dependent on policies and social measures on a large scale. In this type of actions, decisions and policies at the level of public health may be influenced by various factors such as culture, economic, politics, the interests of individuals, etc., but still, any partnership between different government entities and even cooperation with national and international institutions or universities to predict and implement smart measures in the field of cancer can lead to effective results (Puska, 2021).

### Cancer Early Detection

In general, the treatability ratio of cancer is inversely related to the time of diagnosis. In other words, the delay in diagnosis results in difficult treatment. Therefore, according to statistics obtained from comparison of patient survival rates in the early and late stages of the cancer process, it can be inferred that any faster action in early detection and diagnosis of cancer patients can significantly increase the chance of cancer treatability, enhance general health rate, and reduce mortality rate as well (Chen et al., 2020). To achieve these goals, health care providers apply two screening and early diagnosis approaches to seize the initiative in the early detection stage (Organization, 2007).

In a simple expression, the screening approaches refer to a set of methods in which the people with a high risk of cancer or people in the asymptomatic period can be distinguished in a population. Additionally, the identification of precancerous lesions with a high tendency to become cancerous, the presence of cancer, and prevention of the impact of established cancer in a patient are some of the aims of screening methods in the early detection stage. Accordingly, it can be deduced that screening methods can mostly have a part in secondary and tertiary prevention of cancer. Similar to any other methods, screening can also face some challenges in tracking diseases. For instance, screening methods may have less sensitivity and specificity in some cases (Schiffman et al., 2015). Furthermore, some of the screening methods are invasive and can lead to physical damages (e.g., colonoscopy and pap test). In addition to physical damages, patients may experience psychological harm as a result of anxiety before being examined or the pressure and tension from awareness of test results. Along with other disadvantages of screening methods, overdiagnosis and subsequently overtreatment of the disease remains challenging, which may have adverse effects on patients. However, screening recommendations still can be a complementary step towards early intervention and prevention of cancer progression and as one point of view, it is hoped that in the light of the rapid development of technologies, more accurate and efficient screening tests with fewer adverse effects will be available or the efficiency of the current methods will be improved (Wardle et al., 2015).

In addition to screening methods, early diagnosis strategies also play important roles in the early detection of cancer. Early diagnosis strategies can be defined as a set of methods and measures that aim to distinguish symptomatic patients in the early stages of the disease process. To promote this step, applying some strategies such as raising awareness, knowledge, and education in the field of cancer, timely, accurate, and patient-centered diagnosis by the medical doctor to determine the stage of the disease, and providing appropriate therapeutic and practical strategies, are some of the main approaches which have a great contribution to increase treatability and subsequently improve the survival rate, and the quality of life in cancer patients (Organization, 2017).

### Cancer Treatment

In the past, cancer treatment was limited to a few specific methods, such as surgery, and radiotherapy. Since, there was no therapeutic approach to treat systemic diseases caused by metastasis, scientists sought to find a solution to the problem. Therefore, chemotherapy using cytotoxic drugs has been proposed as a suitable solution to increase the long-term recovery rate, which after 60 years is still used as an important and fundamental approach in the treatment of various types of cancer (Crawford, 2013). Although these traditional methods are still common today, they may be associated with different complications (Bidram et al., 2019). For example, although surgery is an essential and important approach in the treatment of cancer, it can have major contribution to the flow of cancer cells in the blood and the formation of metastatic foci. On the other hand, surgery can inactivate anti-tumor immunity and make cancer cells more aggressive which can lead to the recurrence of the disease (Tohme et al., 2017). Hence, in recent years, researchers have sought to take an important step toward improving the treatment rate of cancer patients by applying some of these classical therapies with each other (Im et al., 2016; Zaghloul et al., 2018) or by employing them with new adjuvant therapies like immunotherapy (O'Donnell et al., 2019) and macrophage-based virotherapy (Muthana et al., 2013).

Over the past years, many efforts have also been made to increase insight into tumor biology and cancer progress, which provide a snapshot of more challenges faced by cancer treatment and facilitate the development of more effective strategies in this regard. Previously, cancer was thought to be associated by only changes within cells. However, with the increase of studies on tumors, it has gradually become clear that in addition to intracellular changes, the extracellular alterations caused by tumor micro environment (TME) play an important role in the development of cancer. TME refers to a complex and dynamic structure that encompasses various cell types [e.g., immune cells, fibroblasts, mesenchymal stroma/stem-like cells (MSCs), and adipocytes] located in the extracellular matrix substrate and assist in the progression of the tumor cells. As result of clonal expansion of mutant cells and/or interaction with TME, a phenomenon named tumor heterogeneity emerges, which is not only the main obstacle to the successful treatment of cancers but also can lead to tumor metastasis (Hass et al., 2020). The term of tumor heterogeneity refers to a mixed population of cells which exhibit different molecular signatures in the levels of resistance to therapies, genetic stability, cell surface markers, (epi)genetic alterations, and cell growth, whether within the tumor (inter-tumor heterogeneity) or between tumors (intra-tumor heterogeneity).

In the pursuit to define tumor heterogeneity, some models include stochastic or clonal evolution (CE), the hierarchy or cancer stem cells (CSCs), and plasticity models have been expressed. In the model of CE, the diversification of cells is occurred under the accumulation of genetic and epigenetic changes over time within cells, which can eventually lead to the development of tumorigenic properties in cells. On the other hand, the CSCs model is based on the existence of a small subset of stem cells that provide capabilities such as stemness, self-renewal, and differentiation into cell types that cause tumor heterogeneity. But the plasticity model is based on another belief that can approximately connect the two previous models. Indeed, plasticity is stated that the cancer cells can be transmitted from the stem state to the non-stem cancer cell state and vice versa through reprogramming process (Rich, 2016). Recent studies imply that cell plasticity is a phenomenon that naturally plays an important role in processes such as embryonic development and tissue regeneration. But in the case of cancer, it can lead to the onset, development, and even metastasis of tumor tissue. In a great detail, there are important traces of genetic or epigenetic changes within cells and extracellular changes caused by the cancer microenvironment, behind the cancer cells plasticity. As a result, the populations of CSC–like state cells with capabilities such as self-renewal, immune system evasion, and resistance to chemotherapy can be formed, which can pose many challenges to cancer treatment (Yuan et al., 2019; Hass et al., 2020). With such knowledge, recent studies have focused on discovering more effective ways to increase the rate of cancer treatability while providing a good palliation for patients.

**FIGURE 1:**
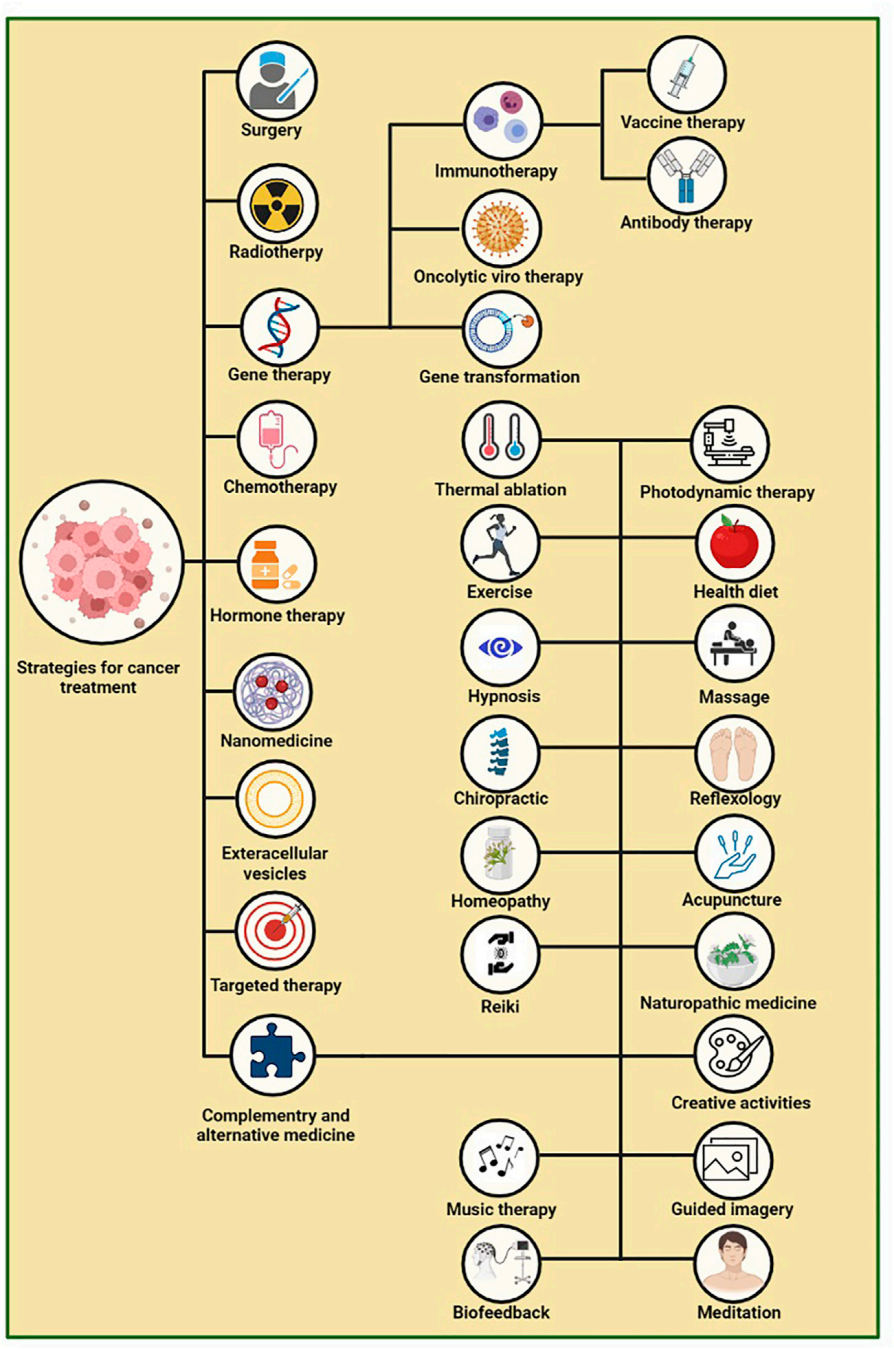
Cancer treatment approaches (Tacón, 2003; Roffe et al., 2005; Jones and Demark-Wahnefried, 2006; Sagar et al., 2007; Lu et al., 2008; Giustini et al., 2010; Masafi et al., 2011; Stanczyk, 2011; Fleisher et al., 2014; Drãgãnescu and Carmocan, 2017; Bilgin et al., 2018; Carlson et al., 2018; Hojman et al., 2018; Yadav et al., 2018; Bidram et al., 2019; Psihogios et al., 2019; Pucci et al., 2019; Laoudikou and McCarthy, 2020; Najafpour and Shayanfard, 2020).

Over recent years, a variety of targeted and alternative methods such as photothermal therapy (PTT), gene therapy, nanoparticle-drug therapy (NDT) (Bidram et al., 2019), extracellular vesicles (EVs), thermal ablation, and magnetic hyperthermia were evaluated for cancer treatment (Pucci et al., 2019). Another group of complementary and alternative medical procedures such as antioxidant systems, exercising (Hojman et al., 2018), acupuncture, yoga, hypnosis, biofeedback, aromatherapy, and massages have been studied, which can be of great help in enduring and coping with the complications of cancer, especially mental and psychological problems (Figure 1) (Singh and Chaturvedi, 2015). However, as recent detailed studies in the field of cancer biology have indicated that molecular alterations at multiple levels including genome, epigenome, transcriptome, proteome, and so on are considered key contributing factors in cancer progression, focus on the field of cancer multi-omics and technologies integrated with this field can be significant step towards facilitating the discovery of novel diagnostic and therapeutic approaches for cancer. Hence in this regard, a better understanding of multi-omics field in cancer needs to be developed that will discuss in more detail in the following section (Charmsaz et al., 2019).

## The Integration of Omics Science With Cancer Research

The word “omics” implies the study of the entire set of molecules in a biological sample. The concept of “omics” consists of broad research areas such as genomics (the study of all genome content), transcriptomics (the study of all RNA transcripts produced by the genome), proteomics (the study of whole proteins products and their interactions), metabolomics (the study of whole metabolites and metabolism processes in a biological sample), and so forth (Arjmand et al., 2021; Esmati et al., 2021; Hosseinkhani et al., 2021; Tayanloo-Beik et al., 2021). Therefore, studying and evaluating biological samples at each omics research areas (Debnath et al., 2010; Omenn et al., 2012) or make a connection between the resultant data and examining them in a multi-omics field can open new doors to investigate and treat the various diseases (Hasin et al., 2017; Misra et al., 2019). In recent years, the omics approaches have also yielded great advances in cancer research. Today, the oncology studies revolve around the idea that molecular changes at different biological layers are inevitable in cancer. Accordingly, omics approaches have been able to provide in-depth insights into the processes involved in cancer, which could decipher cancer’s molecular fingerprint (Menyhárt and Győrffy, 2021).

Genomics is at the most primitive level of the omics branches. Conventionally, the genomics field can provide broad insights into the genome structure, function, and the interrelations between the genes and their products in an organism as well as mapping and editing of the genome. But this importance is heightened when genes and related changes are used to trace the pathology of diseases. Therefore, the use of technologies such as gel electrophoresis, blotting, polymerase chain reaction (PCR), DNA microarray, DNA sequencing, and chromatin immunoprecipitation (ChIP) assay can be effective steps in decoding various aspects of diseases (Falahzadeh et al., 2019). If the meaning of genomics is extended to the field of oncology, a new approach called cancer genomics will find meaning. In a particular define, cancer genomics refers the study of genetic abnormalities related to cancer process which interestingly have an effective contribution in personalized cancer medicine (PCM). Because changes and mutations at the genome level are one of the inseparable facts of cancer, the identification of cancer-specific molecular signatures and mechanisms underlying cancer at the genomic level not only advance the development of the genomics science and related technologies, but also can facilitate the process of cancer management from early detection to treatment (Tran et al., 2012). In this regard, to promote researches, DNA sequencing technologies [from the first generation to the next generation sequencing (NGS)] have opened new doors to the secrets behind the genetic codes of an organism (Mardis, 2019). Traces of sequencing technologies can be found in establishing extensive projects such as the human genome project (HGP) (Hood and Galas, 2003), HapMap (Gibbs et al., 2003), and genome-wide association analysis (GWAS) (Tam et al., 2019) which can be declared as major breakthroughs in human genome studies (Gibbs et al., 2003; Hood and Galas, 2003; Tam et al., 2019). Additionally, in case of cancer, worthwhile projects such as Cancer Genome Project (CGP), The Cancer Genome Atlas (TCGA), and International Cancer Genome Consortium (ICGC) have been developed by applying DNA sequences which focus on the cancer assessment in individuals (Wheeler and Wang, 2013).

Since the advent of genomics, much progress has been made especially in cancer research. One of the advances in this field was the identification of oncogenes [such as RAS family proto-oncogenes (Sugita et al., 2018) epidermal growth factor receptor (EGFR) (Thomas and Weihua, 2019) and Phosphatidylinositol-3-kinase (PI3K)/AKT/mammalian target of rapamycin (mTOR) signaling (Yang et al., 2019)]. More precisely, accurate identification of the role of such oncogenes in the carcinogenic process led to considered these biological components as appropriate drug targets in the treatment of cancers (Sugita et al., 2018; Thomas and Weihua, 2019; Yang et al., 2019). On closer inspection, some studies draw attention to fusion genes (a hybrid gene that is made up of two genes that were previously independent of each other and is now translated and transcripted as a unit) (Parker and Zhang, 2013) and tumor suppressor genes (TSGs) (Wang et al., 2018), including, 1) Caretaker genes refer to the genes which have a fundamental role in preserving genome stability in response to DNA damages (e.g., *BRCA* genes, *PARP1*, NER system, *ATM*), 2) Gatekeeper genes which are responsible for inhibiting proliferation, differentiation and promoting cell death (e.g., *APC*, *RB1*, and *TP53*) (Fanale et al., 2017), and 3) Landscaper genes which are responsible for making products that prepare the fertile environment for cell growth (Macleod, 2000) (e.g., *FGF-2*, *PDGF*, *uPA*) in cancer onset and progression (Andreozzi, 2014). However, in case of disruption in mentioned genes, the cell deviates from regulating state and progresses to a cancerous condition. One of the principles considered in some of the TSG’s dysfunctionality is the “Two-hit” hypothesis. According to this hypothesis, unlike oncogenes, TSGs tend to be recessive. In other words, if one of the alleles of TSGs is inactivated, the normal one can compensate for the dysfunctionality of the mutated allele by producing sufficient products. It is, therefore, necessary to deactivate the function of some TSGs on both alleles to contribute to cancer. However, genetic alterations are not the only cause of the inactivation of TSGs. Indeed, some cellular mechanisms such as ubiquitin-proteasomal degradation, mislocalization of proteins, and aberrant transcription factor regulation can be non-genetic factors affecting the TSGs function, which play an important role in the process of tumorigenesis. Additionally, paying attention to epigenetic factors in parallel with genetic and non-genetic factors is of great importance to lead the “Two-hit” hypothesis moving towards the “Multiple-hit” hypothesis in order to provide broad aspects of tumorigenesis at the molecular level (Wang et al., 2018).

In addition to genetic alterations, the epigenetic regulation is of great importance in cancer researches. Before proceeding to evaluate the effects of epigenetics in cancer studies, it should be provided a general description of epigenetics. In broad biological terms, epigenetics can be defined as heritable mechanisms for regulating genome function which are not attributed to underlying alterations of DNA sequence. In general, the epigenetic phenomena can be developed through mechanisms such as DNA methylation, histone modifications, nucleosome remodeling (Ilango et al., 2020), RNA methylation, and non-coding RNAs, which plays essential roles in modifying the genome function by affecting the structure of chromatin (Lu et al., 2020). To achieve the comprehensive perception of the epigenetic mechanisms landscape, need to zoom in on the genome scale and make our perspective more accurate and detailed. In this context, studies implies that three groups of genes, including epigenetic modulator (e.g., *IDH1/2*, *KRAS*, *APC*), epigenetic modifier (e.g., *SMARCA4*, *PBRM1*, *TET2*), and epigenetic mediator (e.g., *OCT4*, *NANOG*, *LIN28*) are classified as genes related to epigenetic mechanisms that can lead to neoplastic phenotypes in case of disruption (Feinberg et al., 2016). In addition, another point to note is that studies report the effects of epigenetic mechanisms on tumor microenvironment (TME) which is directly involved in the escape of cancer cells from being tracked and destroyed by the immune system (Lu et al., 2020). The interaction of cell environment with epigenetic mechanisms also covers the other aspects. For example, hypoxia is a common phenomenon that occurs in tumor condition due to the high consumption of oxygen by cancer cells with intention to high growth and proliferation. Under hypoxia condition, the oxygen levels are lower than normal. Therefore, the epigenetic mechanisms that require oxygen as an important substance to perform the reaction are disrupted (Camuzi et al., 2019).

Transcriptomics is the next level of omics approaches, which connects the genomics and proteomics levels. In a simple definition, it refers to the study of all transcripts inside cells, which can be analyzed by different methods such as DNA microarray, SAGE, long SAGE, SuperSAGE, HT-SuperSAGE, RNA-Seq, quantitative reverse transcription PCR (RT-qPCR), digital PCR (dPCR), single-cell RNA-Seq, whole exome RNA-Seq, and *in situ* RNA-Seq (Sager et al., 2015). In the field of cancer, the study of transcriptomes can play an essential role in having a comprehensive insight into the mechanisms underlying cancer and discovering valuable biomarkers from various aspects such as alternative splicing, alternative polyadenylation, fusion transcripts, noncoding RNAs, transcript annotation, and novel transcripts (Tsimberidou et al., 2020). In addition to the above, studies suggest that transcriptome studies can provide researchers with valuable information about tumor response to treatment and cancer recurrence or metastasis (Sager et al., 2015).

At the broader level of the omics branches, the proteomics field can be defined, which studies all the contents of the expressed proteins in the cells. Since proteomes are the result of gene expression and are also involved in biological structures and processes, applying proteomics to study cancers can open new doors into discovering novel prognostic, diagnostic, and therapeutic approaches as well as classification of tumors (Kwon et al., 2021). In this regard, methods such as mass spectrometry (MS), enzyme-linked immunosorbent assay, immunoblotting (western blot), and protein microarray can help gain broad insights into cell proteomes and identify processes involved in cancer (Panis et al., 2019). One of the great achievements in the field of cancer proteomics is the decoding of proteomics signatures of 16 types of human cancers, including liver, colon, kidney, esophagus, head and neck, brain, breast, lung, stomach, pancreas, uterus, bladder, prostate, and ovary could pave the way for the use of accurate treatment approaches in different types of cancer by determining the specific type of proteins involved in different cancers (Zhou et al., 2020).

Metabolomics is another branch of omics which generally studies all the metabolites in the body such as hormones, nutrients, drugs, signaling mediators, and the metabolic products in body fluids (Schmidt et al., 2021) and can be measured by different techniques such as nuclear magnetic resonance spectroscopy (NMR), gas chromatography–mass spectrometry (GC-MS), liquid chromatography–mass spectrometry (LC–MS), capillary electrophoresis–mass spectrometry (CE-MS), high performance liquid chromatograph (HPLC), and ultra-performance liquid chromatography tandem mass spectrometry (UPLC-MS/MS) (Goodarzi et al., 2019). What makes the importance of metabolic studies in the field of cancer more prominent, is the widespread effects of mechanisms underlying cancer as well as cancer treatments on the metabolites of the patient (Schmidt et al., 2021). One of these effects is metabolic reprogramming, which play a major role in the development of cancer and in turn can be a suitable goal in the treatment of cancer (Kaushik and DeBerardinis, 2018; Khatami et al., 2019). This phenomenon occurs when cells are exposed to hypoxia and nutrient deficiency due to high proliferation. Hence, the tumor cell adapts to new conditions by shifting the metabolic process from aerobic to anaerobic. Tumor cell reprogramming not only plays a key role in cancer malignancy and metastasis, but also makes the tumor resistant to treatability. Therefore, gaining a comprehensive understanding of the metabolic processes involved in tumorigenesis can be a step towards cancer treatment (Yoshida, 2015).

In addition to the above, in recent years, another area of omics called radiomics has been studied in cancer studies. In general, radiomics is the study of data from imaging processes that can provide appropriate and efficient prognostic and diagnostic information about cancer. Therefore, the information of this branch of omics science can pave the way for accurate identification of the type of cancer and subsequently, the use of appropriate treatment for different cancers (Shur et al., 2021) which can be extracted via different techniques such as magnetic resonance imaging (MRI), computed tomography (CT), and positron-emission-tomography (PET) (van Timmeren et al., 2020). However, the branches of omics studied in the field of cancer are not limited to the mentioned cases and can also cover wide areas of the realm of omics, such as lipidomics (Yan et al., 2018), glycomics (Drake, 2015), pathomics (Gupta et al., 2019), phosphoproteomics (Harsha and Pandey, 2010), immunomics (Basharat et al., 2018), interactomics (Vallet et al., 2021) etc.

One of the most important reasons for omics studies to thrive in recent years, is to save time and money, which has also come with a large amount of data. In addition, molecular alterations within the body are interrelated and multidimensional. Thus, what can bring cancer researchers’ perspectives closer to reality is the integration of data from single omics approaches and expending it into a vast realm called multi-omics. What makes the multi-omics data from cancer cells debate so valuable is that the multi-omics data analysis can provide the possibility of 1) clustering multiple biological contexts, 2) unraveling the complexities of genotype-phenotype-environmental interactions, 3) identification of cancer-associated phenotypes for timely prognosis and prediction, 4) investigating the effects and aspects of the applying therapeutic approaches, and 5) facilitating the bench to bedside studies by creating models that mimic the complexities of biological condition (Menyhárt and Győrffy, 2021).

## ML, a Novel Computational Technology in Biological and Medical Researches

Over the past years, the advent of novel technologies in the field of medical researches has revolutionized the study of various aspects of diseases and led to the emergence of a field called medical technology. In a simple expression, medical technology refers to the arrival of advanced tools and innovations into the health system to redrawing the healthcare landscape, push the boundaries of how target health issues, and solve the problems to promote health status of individuals or even the society. Therefore, medical technologies are without doubt one of the key markets of the near future (Ortiz and Hsiang, 2018). In the study of medical technologies, researches come across a vast realm called AI (Cosgriff et al., 2020). In general terms, AI is a technology derived from computer science and mathematics, which aims to simulate of natural intelligence carried out by machines with intention to learn and mimic human-like tasks (Cipolla-Ficarra et al., 2021). Over the past years, the entry of AI into the fast-growing field of medicine has been a key step towards health promotion and disease management through both virtual and physical aspects. In discussing the physical aspect of AI, the role of robotics in helping surgeons and people with disabilities can be mentioned. But in the other aspect, scientists are dealing with virtual assistants that have rushed to the aid of the health care system. In this context, ML, as a branch of AI, has gained increasing interest as one of the novel computational technologies in scientific researches over recent years (Hamet and Tremblay, 2017).

In general term, ML, refers to a subset of AI which aims to analyze input data, obtain patterns between data, and make predictions for output data based on systematic algorithms through the integration of statistics, mathematics, and computer science (Sidey-Gibbons and Sidey-Gibbons, 2019). Studies imply that the term “Machine learning” was first coined by Arthur Samuel in 1959 to study the game of checkers. According to the reports, Samuel believed that if computers were programmed, they could play checkers. In this context, he represented two methods including, “Rote learning” and “Generalization learning” to advance the mentioned goal. Thus, these two methods formed the basis of one of the first working programs in AI realm (Samuel, 1959). Since then, many studies have been conducted in ML field which have been accompanied by the development of different approaches and techniques (Minsky and Perceptrons, 1969; Werbos, 19741974; Quinlan, 1986; Cortes and Vapnik, 1995; Breiman, 2001). To promote research in this field toward, this review firstly highlights the main approaches to get acquainted with how the technology of ML exactly works.

Basically, the major ML approaches can be discussed under four headings including, Supervised learning, Unsupervised learning, Semi-supervised learning, and Reinforcement learning which are as detailed below (Sedghi et al., 2020).

### Supervised Learning

Supervised learning is the most popular pattern for performing ML operations and is widely used where there is an accurate mapping between input-output data. In other words, supervised learning initially begins with importing datasets include training attributes and target attributes. The supervised learning algorithm obtains the relationship between the training examples and their specific target variables. When these steps have been completed, the system uses that learned patterns to categorize completely new inputs (Cunningham et al., 2008). Classification and Regression are two types of supervised learning tasks which are selected according to datasets. In a simple expression, the goal of classification task is to predict discrete values. However, if the data set is continuous values, the regression task is used for supervised learning. In a comprehensive review of this type of learning, in addition to tasks, supervised learning techniques such as support vector machine (SVMs), neural network, naive Bayes, logistic regression, memory-based learning, decision trees, random forests, bagged trees, boosted trees, and boosted stumps should also be considered (Nasteski, 2017).

### Unsupervised Learning

In unsupervised learning, the data is not predetermined and the system deals with unlabeled data. In other words, the input variable is given without any corresponding output variable. Hence, a model is prepared through self-training process of algorithms in which unknown patterns and information are discovered without receiving information from the environment or teacher guidance. Clustering, association, and dimensionality reduction are among the techniques used in this approach (Ghahramani, 2003; Hastie et al., 2009). In clustering technique, the main idea is zoomed in finding the pattern with intention to divide the data into several groups with common attributes (Deepak, 2016). This is while, the association technique deals with discovering relationship among variables which can have a great contribution to discover knowledge from a data set (Cios et al., 2007). Moreover, real-world data may have multi-layered variables and a large number of attributes, so-called high-dimensional data, an example of which can be found in the data obtained from fMRI scans. This issue can make ML processing face many problems. Therefore, it is tried to reduce data dimensionality to facilitate the processing data by applying dimensionality reduction patterns (Van Der Maaten et al., 2009).

### Semi-Supervised Learning

In a simple expression, semi-supervised learning is a combination of unsupervised and supervised learning. In other words, this approach uses a small set of labeled data along with a large amount of unlabeled data to improve ML task performance which is a more relevant scenario for costly and rare labeled data. Additionally, in ML approaches, the use of semi-supervised learning has received considerable attention. What distinguishes this type of data processing is that in both human learning and semi-supervised learning, most of the input data is unlabeled and the success of the input data processing depends on some assumptions. Hence, this type of learning is closely related to human learning (Zhu and Goldberg, 2009).

### Reinforcement Learning

**FIGURE 2:**
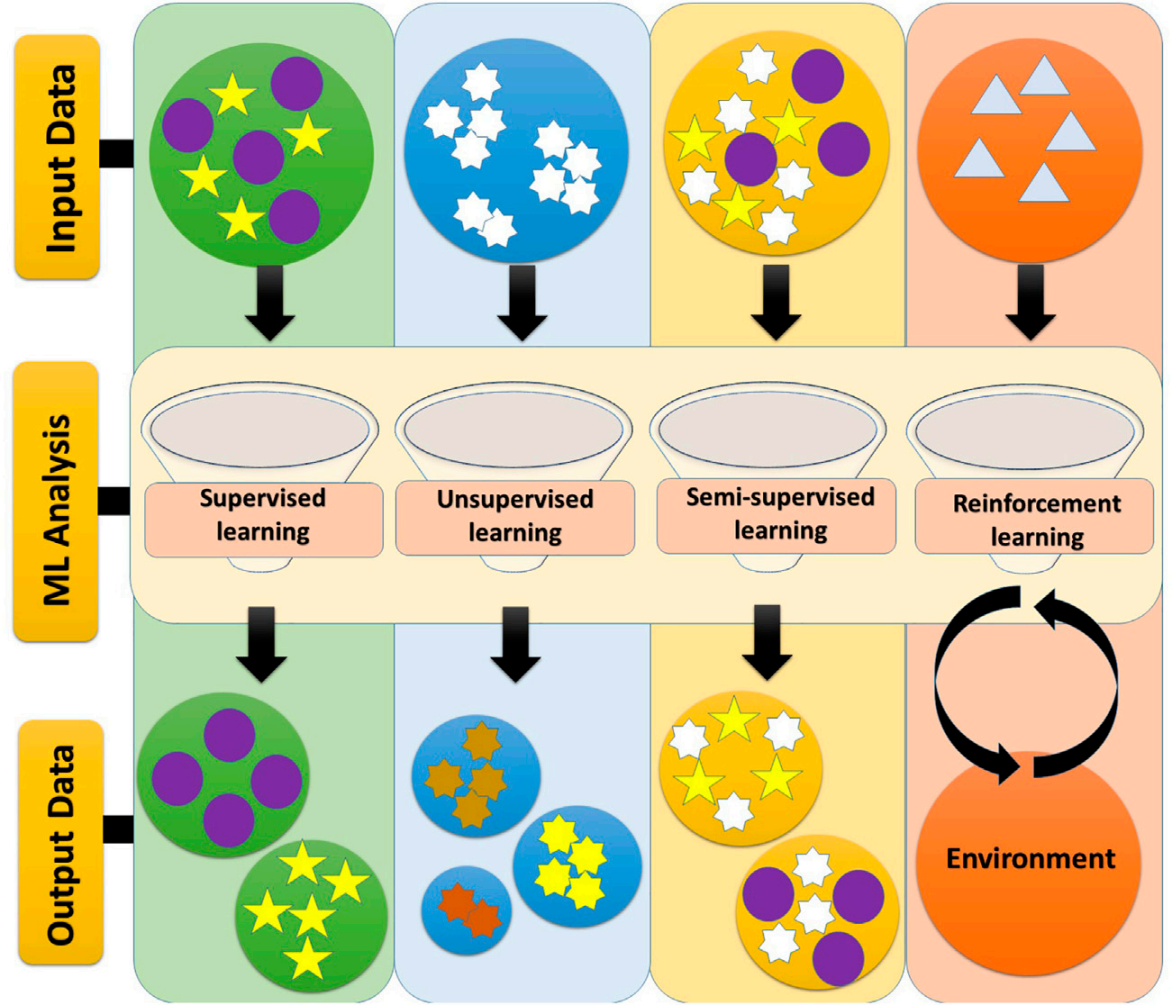
ML approaches. The main approaches of machine learning include: 1) Supervised learning, 2) Unsupervised learning, 3) Semi-supervised Learning, and 4) Reinforcement learning. In supervised learning, the input and output are specified and the data is labeled. In unsupervised learning, specific data does not already exist and is not intended to be an input-output connection, but only to categorize them. Semi-supervised learning uses both labeled and unlabeled data simultaneously to improve learning accuracy. Reinforcement learning loop has a sequence of modes, actions, and rewards (Sedghi et al., 2020).

In this type of learning approach, there is an interaction between the two elements of the environment and the learning factor. Indeed, reinforcement learning loop has a sequence of modes, actions, and rewards. In this regard, the goal of the agent is to maximize the expected (cumulative) storage reward (meaning expected is mathematical hope) (Figure 2) (Sutton and Barto, 1999; Woergoetter and Porr, 2008).

## ML in Multi-Omics Data Analyses of Cancer

In recent years, the advent and development of computing technologies has targeted the heart of disease biology in precision medicine. Therefore, it has been promising the emergence of a new generation of diagnostic and therapeutic approaches. Meanwhile, the integration of omics branches with computing technologies holds further promise to unravel the mechanisms underlying the biological condition of interest and enhance scientific understanding of the detailed roles of complicated cellular interactions in human diseases. In this field, precision medicine includes two important discussions of the source for data production and the use of data obtained for modeling (Tebani et al., 2016). In cancer researches, precision medicine can deal with resources such as huge data from cancer studies in various branches of omics to use them for diagnostic and therapeutic purposes. In this case, the introduction of computing technology, such as ML, as a branch of AI can take on the task of modeling this huge data, which can open new doors to the comprehensive perception of the cancer landscape (Nicora et al., 2020). Therefore, in the pages that follow, this paper will review the research conducted on the application of ML approaches and techniques in the field of cancer multi-omics analysis.

In 2015, Emaminejad et al. conducted investigations in the field of predicting recurrence of stage 1 non-small-cell lung cancer (NSCLC) after surgery by integrating ML methods with two genomics and radiomics branches. For this purpose, they use computed tomography (CT) imaging 79 cancer patients as the radiomics data source. On the other hand, two biomarkers including the excision repair cross-complementing 1 (ERCC1) genes and a regulatory subunit of ribonucleotide reductase (RRM1) were also used as the basis of genomics data in this study. Then, they applied computer-aided detection (CAD) and radiologists’ assessments to classify lung tumors. To promote the study, they trained naïve BN classifier using eight radiomics features and a multilayer perceptron classifier using two genomic biomarkers. As a result of this study, the area under a curve (AUC) values were reported 0.78 ± 0.06 in the case of raidomics features application and 0.68 ± 0.07 in case of geneomics biomarkers application. This is while the merger of these two omics branches was able to increase the AUC values to 0.84 ± 0.05. Therefore, it can be concluded that although the ratio obtained radiomics classification was more than genomics case, the integration of these two branches can be a promising approach to predict NSCLC in patients (Emaminejad et al., 2015).

A year later, Yu et al. focused their studies on the analysis of genomics, transcriptomics, and proteomics profiles of patients in integration with ML methods to investigate the molecular and morphological alterations involved in lung adenocarcinoma cancer. In this study, they initially processed the digital whole-slide histopathology images of the 538 patients and obtained the statistical data needed from the images in parallel with the reports obtained from patients to determine the stage of cancer. In the next step, they analyzed the data by extracting the genomics, transcriptomics, and proteomics profiles from the TCGA and Cancer Genome Atlas Research databases in integration with the Breiman’s random forest method of ML. Finding correlations between the degree of cancer pathology and genomics or proteomics profiles using ML methods was one of the goals of the researchers to investigate the effective mechanisms in tumorigenesis process. They also considered the correlation of quantitative histopathological features with TP53 mutation and histology of sub-classifications of lung cancer. Then, they developed regularized Cox proportional hazards models using multi-omics and histopathology data as well as patient age to predict patient survival rates. Therefore, it can be concluded that the study of Yu et al. is one of the examples of efficient studies which show the effect of integrating the data obtained from several sources to predict the cancer (Yu et al., 2017).

In another study, the use of entitled fast-multiple kernel learning framework (fMKL-DR) as one of the ML methods, was presented by Giang et al. to address the challenges of big data obtained from multi-omics studies in Alzheimer’s disease (AD) and cancer patient stratification. In this regard, sets of data (genomics and radiomics data for AD patients and genomics and proteomics data for cancer patients) were initially needed. For this purpose, in the case of AD, genomic data as well as MRI images as radiomics data were extracted from the Alzheimer’s Disease Neuroimaging Initiative (ADNI). Whereas, the genomics and proteomics data needed to study cancer patients such as gene expression, DNA methylation, and miRNA expression were obtained from the TCGA database for six types of cancer, including 1) Squamous cell lung carcinoma, 2) Breast invasive carcinoma, 3) Glioblastoma multiforme, 4) Ovarian serous cystadenocarcinoma, 5) Liver cancer, and 6) Kidney renal clear cell carcinoma. After analyzing the data, preprocessing and optimizing processes have been performed to reduce the problems such as the existence of noisiness, data redundancy, and missing data. Then, kernel matrices were created, which were integrated using multi-kernel learning framework to create a comprehensive and general matrix. These were the processes that ultimately led to the creation of a predictive binary classification model with support-vector machines (SVM). In the case of cancer patients, it can be concluded that the use of different types of data could greatly increase the accuracy of the classification model compared to the use of one type of data. More precisely, the integration of the data caused the accuracy of the model to reach a range of about 72–94%. In addition, the results of the study indicated that high accuracy has been linked to breast (94.29%) and kidney cancers (87.50%), respectively (Giang et al., 2020).

Another study conducted in 2020, examined the use of ML methods in the analysis of Acute lymphoblastic leukemia (ALL) data. In this study, Li et al. extracted data related to gene expression and DNA methylation from Gene Expression Omnibus (GEO) database. After analyzing the obtained data by Boruta and Monte Carlo feature selection methods, they studied the differences of the two types of cancer, named BCP-ALL and T-ALL, which was accompanied by important results. For example, as a result of these studies, 7 expression signature genes and 175 methylation signature genes were obtained, in which two genes, including *CD3D* and *VPREB3* were common. In addition, it has been implied that *CD3D* gene has a major regulatory contribution in the cell and molecular process of this type of cancer (Li et al., 2020).

In 2020, the integration of deep learning-based autoencoding method with various omics fields, including genomic, transcriptomics, and epigenomics (mRNA, miRNA, DNA methylation, and copy number variations) involved in lung adenocarcinoma was first carried out by Lee et al. In this study, the aim was to achieve a risk classification model for patients with lung cancer in order to accelerate the prognosis of the disease in the early stages. It is also worth mentioning that in this study, the data obtained from the integration of omics approaches were considered as training variables and the data of each layer of omics field were solely analyzed to confirm the created model. Accordingly, regarding training variables, TCGA was used as a multi-omics database. However, both TCGA and Gene Expression Omnibus (GEO) databases were used to collect variables for validation process. After analyzing the input data to Autoencoder, survival features and survival subgroups were evaluated by Univariate Cox regression, Lasso regression, and K-means clustering methods and algorithms. Next, the strength of a statistical model was validated using random forest algorithm along with the independent data obtained from each single omics branches. As in previous studies, this study indicated that the accuracy of the model resulting from the integration of omics data is better than the accuracy of the data from analyzing each branch of omics by deep learning-based autoencoding method. The study also reported that adding patients’ clinical and personal information to available data could increase accuracy and greatly improve model performance (Lee et al., 2020).

In 2021, Zhang et al. also included their study in introducing the deep learning methods into the field of multi-omics data analysis of cancer. This study aims to evaluate the multi-omics data of muscle-invasive bladder cancer (MIBC) based on deep learning-based autoencoding method with intention to improve prognostic approaches for this type of cancer. For this end, the data related to gene expression, gene copy number variations, miRNA expression, and DNA methylation was extracted from TCGA-MIBC dataset and used them as input data to autoencoders. As in the previous study, the data were categorized as training and validating data. In addition, algorithms such as random forest, Naïve Bayes, k-Nearest Neighbor, and Adaboost were employed in data processing stage. As a result of these analyzes, the data were divided into two subtypes high-risk and low-risk patients, which yielded significant results from the comparison of these two. For instance, genomic and immunomics differences were observed in these two groups. Also, the activity of signaling pathways and biochemical processes related to the disease was observed more in the high-risk group than in the other group. And finally, the remarkable point about this study was the recognition of KRT7 as a biomarker of MIBC (Zhang et al., 2021). However, the application of deep learning methods is not limited to this type of cancer. In this regard, some papers such as the study by Hira et al. recently demonstrated that the use of deep learning-based autoencoding method can be considered a practical approach in the multi-omics data analysis of ovarian cancer (Hira et al., 2021).

In addition, some studies demonstrated that ML approaches have held further promise to enhance our understanding of the prognosis of chemotherapy success for cancer patients. For instance, in 2018, Borisov et al. evaluated the efficiencies of anticancer drugs on cancer patients by transferring the data from drug-treated cell line gene expression datasets to the small cases of patients in combination with ML approaches. In this regard, the categories of cell lines based on their IC50 values were applied in parallel with the gene expression profiles as cell line data. Regarding patients, gene expression profiles and treatment outcomes were also used as the other source of experiments data. To promote the research, the leave-one-out procedure and AUC metric with a predefined threshold were applied to investigate the features obtained from gene expression datasets. In addition, three ML methods including, support vector machines, binary trees, and random forests were employed to analyze the data with more validity, in parallel with two previous methods. Therefore, it can be concluded that the use of data obtained from gene expression profiles of cell lines and their drug treatments outcomes in the integration of ML approaches, can be a promising tool to develop effective personalized medicine approaches for cancer patients (Borisov et al., 2018).

However, the prognosis of chemotherapy success for individual cancer patients by ML approaches is still far from the ultimate solution, due to essential deficiency of preceding cases (i.e., patients with known response to certain a type/regimen of treatment and corresponding multi-omics profiles), which can lead to extrapolation during ML application. Hence several studies have been recently focused on addressing the possible extrapolation problems in the application of ML methods in the prognosis of cancer chemotherapy responses. For instance, in 2018, Borisov et al. highlighted the need to remove irrelevant features from datasets to decrease the extrapolation. By drawing on the concept of avoiding extrapolation, Borisov represented the application of flexible data trimming (FDT) procedures such as floating window projective separator (FloWPS) to improve the performance of global ML methods in increasing the quantity and quality of prognostic biomarkers, which have a major contribution to monitoring the chemotherapy treatment responses (Borisov and Buzdin, 2019).

Similarly, in 2020, Tkachev et al. provided an in-depth analysis of the work of FloWPS to enhance the efficiency of ML methods in personalized cancer medicine based on omics data analysis. This method, which is based on preventing the occurrence of extrapolation in the feature space and increasing the feature importance correlation, was examined on seven methods of ML, including 1) Linear SVM, 2) Random forest, 3) Binomial naïve Bayes, 4) Adaptive boosting, 5) Multi-layer perceptron, 6) Tikhonov (ridge) regression, and 7) *k* nearest neighbors. The application of FloWPS not only increased the quality and efficiency of the first five mentioned approaches of ML but also caused an increase in the AUC rate related to the treatment response of 1,778 cancer patients. It should also be noted that among the mentioned methods, the integration of FloWPS to binomial naïve Bayes method has performed remarkably well in data trimming (Tkachev et al., 2020).

Additionally, in 2021, Borisov et al. also demonstrated that multiple myeloma (MM) patients act differently in response to bortezomib (the inhibitor of the proteasome enzyme complex within the cell) as one of the fundamental chemotherapy approaches. Therefore, the authors tried to find prognostic biomarkers to take a step toward targeted treatment of MM patients by creating RNA sequencing profiles of three groups of patients treated by bortezomib, doxorubicin, dexamethasone (PAD), and bortezomib, cyclophosphamide, dexamethasone (VCD), or treated by the combination of both PAD and VCD, respectively. In this study, the patients’ profiles had been divided into two groups of good and poor responders by applying ML algorithms. In this context, five ML methods, including support vector machines (SVM), Tikhonov (ridge) regression (RR), binomial naïve Bayes (BNB), random forest (RF), and multi-layer perceptron (MLP) were developed of which BNB method had the best performance for determining the PAD + VCD cohort and MLP method was an optimal solution for the VCD cohort. Additionally, it should be noted that in both successful results, FloWPS dynamic data trimming method was used as a practical approach to transforming data from a high-dimensional space into a low-dimensional space. Furthermore, the RNAseq and microarray datasets results implied that in both groups of good and poor responders, five genes, including *FGFR3, MAF, IGHA2, IGHV1-69*, and *GRB14* were overexpressed (Borisov et al., 2021).

If the perspective of cancer management extends from a single disease to the management of different types of cancer, it becomes clear that some therapeutic approaches, such as radiotherapy, still face significant challenges. Therefore, recently, Lewis et al. demonstrated that the use of ML methods and algorithms in integration with multi-omics data analysis of cancer can be a beneficial approach to identify some biomarkers in evaluating radio sensitivity of tumors. Since data related to tumor metabolomics are not widely available, methods such as flux balance analysis (FBA) can have a great contribution to address this problem. Therefore, in this study, the personal FBA model is applied, which was developed by the integration of genomic, transcriptomic, kinetic, and thermodynamic data from the aggregation of 716 radiation-sensitive data and 199 radiation-resistant patient tumors. This model was then analyzed in combination with ML classifiers. As a result of this study, the high accuracy (AUC about 0.906 ± 0.004) was reported in the model used to integrate data from different omics fields. In addition, this method played an important role in identifying subgroups of patients as well as metabolic biomarkers in resistance to ionizing radiation (Lewis and Kemp, 2021).

In the end it should be stated that the application of ML methods and algorithms goes far beyond what is stated in this article and can cover a wide range of cancers such as liver (Chaudhary et al., 2018), prostate (Wang et al., 2021a), colorectal (Kammonah, 2021), and premenopausal breast cancer (Fröhlich et al., 2018).

## Challenges

When the data analysis goes beyond a branch of omics science and is examined in several layers of multi-omics field, the process of data analysis faces many challenges due to the creation of a vast and extensive source of data with intricate communications. Therefore, being aware of these challenges as well as being familiar with some possible solutions to solve them can pave the way for the desired goals (Table 1) (Picard et al., 2021).

**TABLE 1:** Challenges of multi-omics data analysis by ML and their solutions.

Challenges	Consequences of the challenge	Solution	References
Complex data sets with a large amount of additional meaningless information in it	Existing patterns can difficultly be analyzed or described in the vast amount of omics data layers. In addition, classification accuracy will be decrease and the prediction of meaningful data will be difficult	1) Ensemble techniques	Picard et al. (2021), Gupta and Gupta, (2019), Caiafa et al. (2021)
		2) Distance based algorithms	
		3) Single learning based techniques	
		4) Deep learning method based on an auto-encoder architecture	
		5) EMD method	
		6) dubbed ELCs	
High number of omics data variables compared to the study sample	Data dimensionality increases (curse of dimensionality)	Dimensionality reduction methods include:	Picard et al. (2021), Mirza et al. (2019)
		1) Linear FE methods such as PCA, MCIA, joint NMF, and MOFA	
		2) Nonlinear FE methods such as t-SNE, autoencoders, and representation learning	
		3) Filter methods of FS technique such as mRMR, FCS, Information Gain, and ReliefF	
		4) Wrapper methods of FS techniques such as RFE-SVM, Boruta, and jackstraw	
		5) Embedded methods of FS technique such as LASSO, Elastic Net, and stability selection	
Data heterogeneity (Data with different types or different distributions)	The balance of ML is upset and data integrity is prevented	If there is naive feature concatenation-based data integration:	Picard et al. (2021), Mirza et al. (2019)
		1) Tree-based methods such as decision trees and random forest	
		2) penalized linear models such as Elastic net, LASSO, and TANDEM	
		If there is simple feature concatenation-based integration	
		1) MKL methods such as simple MKL and Bayesian multitask MKL	
		2) Graphs and networks methods such as SNF, NetICS, PARADIGM, and HetroMed	
		3) Latent sub-space methods such as iCluster+, Scluster, and MV-RBM	
		4) Deep learning methods such as multimodal DBN, multimodal DNN, improved CPR, and AuDNNsynergy	
Class imbalance	It can lead to increase in the degree of overlapping among the classes and limit the size of training data. In addition, class distributions become highly imbalanced. If the balance within a class is lost then a small disjuncts is appeared	1) Data sampling methods such as: under sampling the majority class algorithms, oversampling the minority class algorithms, and combination of both under sampling the majority class and oversampling the minority class algorithms	Picard et al. (2021), Mirza et al. (2019), Ali et al. (2013)
		2) Cost-sensitive learning methods such as Mnet, UNIPred, SVM_weight, and Spotlite	
		3) Ensemble methods such as Balanced Cascade, EasyEnsemble, ensemble with WMV, and WELM	
		4) Evaluation measures methods such as Diablo, SNN, WMV, and FPRF	
Missing data	It can lead to increase in parameters bias and complexity of the analysis and reduction in representative sample and statistical power	If there are sufficient amounts of sample:	Picard et al. (2021), Mirza et al. (2019), Kang, (2013)
		1) Listwise deletion	
		In the other cases:	
		1) Matrix factorization methods such as ALRA, SVD-impute, and SparRec	
		2) Autoencoders methods such as MIDA, multilayer autoencoder, and AutoImpute	
		3) Integrative imputation methods such as MOFA, LF-IMVC, and ensemble regression imputation	
		4) Maximum likelihood approaches such as EM algorithm and Direct Maximization	
		5) Single imputation methods such as replacement with mean or mode values, hot-deck imputation, regression imputation, and k-nearest neighbor	
		6) MI methods for liner analysis such as MI-MFA, MCMC, and MICE	
		7) MI methods for non-liner analysis such as MICE with RF, MIDA, and GMM-ELM	
Data scalability	Practical data processing workflow for multi-OMICS projects based on ML approaches becomes difficult and problematic on a single computer	1) Efficient algorithms for big data such as non-iterative neural networks, scalable MKL methods, and convex optimization for big data	Mirza et al. (2019)
		2) Online training algorithms such as OS-ELM, IDSVM, and online deep learning	
		3) Distributed data processing methods such as Spark’s MLlib, Apache Mahout, and Google’s Tensor Flow	
		4) Cloud computing-based solutions such as Galaxy Cloud, MetaboAnalyst, XCMS online, Omics pipe, and ML-as-a-service	

ALRA, Adaptively-thresholded low-rank approximation; AuDNNsynergy, Deep Neural Network Synergy model with Autoencoders; Diablo, data integration analysis for biomarker discovery using latent components; ELCs, embedding label correlations; ELM, extreme learning machine; EM, expectation-minimization; EMD, empirical mode decomposition; FCS, correlation-based FS; FE, Feature extraction; FPRF, fuzzy pattern random forest; FS, Feature selection; GMM, Gaussian mixture model; IDSVM, incremental and decremental support vector machine; improved CPR, improved Clustering and PageRank; Joint NMF, Joint non-negative matrix factorization; KRR, kernel ridge regression; LASSO, least absolute shrinkage and selection operator; LF-IMVC, Late Fusion Incomplete Multi-View Clustering; MCIA, Multiple co-inertia analysis; MCMC, Markov-chain Monte Carlo; MI, Multiple imputation; MICE, multivariate imputation by chained equation; MIDA, denoising autoencoder-based MI; MI-MFA, MI for multiple factor analysis; MKL, Multiple kernel learning; ML, Machine learning; MOFA, Multi-omics factor analysis; mRMR, maximal-relevance and minimal-redundancy; multimodal DBN, multimodal deep belief networks; multimodal DNN, multimodal deep neural networks; MV-RBM, mixed variable restricted Boltzmann machine; NetICS, Network-based Integration of Multi-omics Data; OS-ELM, online sequential extreme learning machine; PARADIGM, PAthway Recognition Algorithm using Data Integration on Genomic Models; PCA, Principal component analysis method; RF, random forest; RFE-SVM, recursive feature elimination-support vector machine; SNF, similarity network fusion; SNN, super-layered neural network architecture; SparRec, Sparse Recovery; SVD, singular value decomposition; SVM, support vector machine; t-SNE, t-distributed stochastic neighbor embedding; UNIPred, unbalance-aware network integration and prediction of protein functions; WELM, weighted extreme learning machine; WMV, weighted majority voting.

## Conclusion and Future Perspective

Cancer, as a world wild serious disease, can affect various biological layers of the human body. Due to the complex mechanisms involved in cancer, the use of the realm called omics and its various branches has recently attracted the attention of many researchers. Since the interaction between the biological processes involved in cancer is complex and occurs in multiple layers, the study of the mechanisms involved in different types of cancer requires molecular studies in several layers of omics called multi-omics field. Due to the high volume of data in the realm of multi-omics, the application of computing technologies such as ML has been highlighted. Today, ML algorithms and method was heralded as a major breakthrough and a pioneer approach in the analysis of cancer multi-omics data with intention to the prognosis, diagnosis, classification, and identification of biomarkers. Therefore, it can be promising to discover more effective and accurate diagnostic and therapeutic approaches to manage and control cancer growth in future generations (Biswas and Chakrabarti, 2020).

In recent years, a variety of studies aimed to classify patients and identify cancer biomarkers using ML methods in integration with multi-omics data analysis (Nicora et al., 2020; Wang et al., 2021a; Gallardo-Gómez et al., 2022). However, Wang et al. went one step further and suggested MOGONET as a supervised multi-omics integration framework to facilitate data analysis and data classification. In a comparative study, MOGONET was more effective and efficient than the integrated multi-omics methods in performance classification tasks. In addition, MOGONET can efficiently perform cross-omics correlations and omics-specific learning via employing view correlation discovery network (VCDN) and graph convolutional networks (GCN), respectively. Therefore, they can perform their classification task well. Additionally, MAGONET can be used for a variety of omics data types. Moreover, in most cases, MOGONET does not react much to the change of the *k* parameter, which can show the superiority of this framework well. Therefore, this framework can be a very effective approach to diagnose different biomarkers and pave the way for the discovery of treatment approaches in different types of cancers (Wang et al., 2021b).

Another breakthrough in the integration of computing analysis with the omics field is the introduction of an algorithm called QueryFuse. The development of this algorithm followed that RNA-seq approach, which can be used as one of the favorite platforms for examining the fusion of genes, is costly and time-consuming. The development of the QueryFuse algorithm is a great breakthrough to solve the existing problems and facilitate the identification of gene-specific fusion from pre-aligned RNA-seq data. In addition to identifying all isoforms of hypothesized fusions and most of the experimentally validated fusions in data sets, the QueryFuse algorithm was able to detect the highest recall rate (90%) and precision rates (99%) in the data sets compared to TopHatFusion and defuse algorithms. Hence, this algorithm shows high superiority over the other two algorithms in both real and simulated data sets (Tan, 2020).

Another important issue in diagnosing diseases such as cancer is identifying the copy number variants (CNVs), Studies have shown that CNVs play a key role in cancer metastasis, which can be obtained through exomes of circulating tumor cells and cell-free DNA (cfDNA). However, data obtained from genomic studies are abundant and current methods are not completely reliable. Hence, the use of bioinformatics approaches and algorithms such as Wisecondor WisecondorX, ExomeCNV, SAvvyCNV, MFCNV, VarScan 2, ADTEx, and CNV_IFTV can facilitate the study of the obtained data (Pös et al., 2021).

In addition, recently, traces of the entry of ML into the field of multi-omics data analysis from neuro-oncology research have been seen. So that the application of this advanced technology in various fields of cancer is getting more colorful and effective (Takahashi et al., 2021).
